# Schiff Base Hydrogel Bio-Adhesive Using Oxidized Chondroitin Sulfate and Polyethylenimine with Antibacterial Properties and Cytocompatibility

**DOI:** 10.3390/polym18172091

**Published:** 2026-08-28

**Authors:** Lei Nie, Mengqing He, Xiaoran Hu, Zihan Sun, Yingying Liang, Shichang Cheng, Ling Wang, Mengke Chen

**Affiliations:** 1 College of Life Sciences, Xinyang Normal University, Xinyang 464000, China; 2 Dabie Mountain Laboratory of Henan Province, Xinyang 464000, China; 3 School of Medicine, Xinyang Normal University, Xinyang 464000, China

**Keywords:** hydrogels, polyethylenimine, chondroitin sulfate, bio-adhesives

## Abstract

Hydrogel bio-adhesives have gained great attention in wound healing and tissue regeneration applications because conventional wound closures are often hindered by insufficient adhesion and poor biocompatibility. Considering that dynamic covalent interactions facilitate robust wet adhesion in hydrogels, we fabricated a Schiff base hydrogel bio-adhesive based on oxidized chondroitin sulfate (OCS) and polyethylenimine (PEI), and employed different degrees of OCS oxidation to regulate the physicochemical properties of the bio-adhesives. In this system, aldehyde groups of OCS react with amino groups of PEI to form covalent imine crosslinks, while physical hydrogen bonds also contribute as supplementary interactions. The fabricated hydrogel bio-adhesives demonstrated a three-dimensional interconnected microstructure and regulated equilibrium swelling ratios. The rheological tests also confirmed the typical viscoelasticity of the hydrogels and their shear-thinning behavior. The obtained hydrogel bio-adhesives demonstrated rapid and autonomous self-healing ability and strong adhesion to the surfaces of various matrices and wet organs, including wood, glass, metal, plastic, rubber, and heart, liver, spleen, stomach, and lung tissue. Furthermore, an ABTS radical scavenging assay confirmed their potent antioxidant activity. The hydrogels possessed effective antibacterial activities against Gram-positive *Staphylococcus aureus* and Gram-negative *Escherichia coli*. The hydrogels exhibited good hemocompatibility, effective intracellular reactive oxygen species (ROS) scavenging activity, favorable cytocompatibility, and promoted cell proliferation. These results confirmed the fabricated hydrogels via Schiff base connections for biomedical applications and provided a facile design for biomedical hydrogel bio-adhesives.

## 1. Introduction

Hydrogels have attracted considerable attention in wound dressings, tissue engineering, and drug delivery. This is attributed to their three-dimensional porous networks, high water content, and extracellular matrix-like microenvironment [1,2,3]. However, conventional hydrogels often exhibit weak mechanical strength, limited biofunctionality, and poor ability to regulate the wound microenvironment [4,5]. These drawbacks prevent them from meeting the integrated demands of hemostasis, antibacterial activity, anti-inflammation, and cell proliferation in complex wound repair [1,6,7]. Therefore, multifunctional hydrogels fabricated from natural polysaccharide derivatives and functional polymers—featuring tunable mechanical properties, dynamic self-healing capacity, and multiple biological activities—have become an important research direction in wound repair materials [8,9].

Chondroitin sulfate (CS) is a natural sulfated glycosaminoglycan composed of alternating D-glucuronic acid and N-acetyl-D-galactosamine residues linked via β-1,3 and β-1,4 glycosidic bonds. As an essential constituent of the extracellular matrix, CS possesses good biocompatibility, biodegradability, and biological activities including anti-inflammatory and antioxidant effects [10,11,12]. However, CS lacks reactive groups for rapid crosslinking and has high water solubility, making in situ gelation difficult [13]. To address this, oxidized chondroitin sulfate (OCS), which is CS bearing reactive aldehyde groups generated by selective oxidation of its vicinal diol groups, has been prepared.

OCS retains the beneficial properties of CS and has aldehyde groups for Schiff base crosslinking with amino groups of polymers. This chemical crosslinking strategy enables rapid in situ gelation of hydrogel networks under physiological conditions and imparts dynamic reversibility, self-healing properties, and injectability [14,15]. For example, an OCS/gelatin hydrogel exhibited a highly porous structure, favorable water retention, and good hemocompatibility, showing its potential as a wound dressing [16]. Introducing N, O-carboxymethyl chitosan further endowed OCS hydrogels with injectable, self-healing, antibacterial, and hemostatic properties, contributing to enhanced wound healing [15]. Moreover, integrating dynamic thiol-aldehyde addition into CS/hyaluronic acid networks enabled rapid gelation and on-demand dissolution, offering an alternative approach for dynamic wound management [13].

Polyethylenimine (PEI) is a water-soluble cationic polymer with a high density of protonated amino groups. Its proton sponge effect and positive charge enable both wet-tissue adhesion and broad-spectrum antibacterial activity (via membrane disruption) in the wound microenvironment [17,18,19,20]. Kohlan et al. prepared a rapidly forming hydrogel based on PEI and oxidized hyaluronic acid, which showed strong tissue adhesion to wet wound surfaces [21]. Fang et al. prepared a hydrogel by physically crosslinking carboxymethyl chitosan and PEI. The incorporation of PEI enhanced the antibacterial activity and improved wet-tissue adhesion, suggesting its potential for skin wound healing [17]. Nie et al. prepared a rapidly forming adhesive hydrogel based on lactobionic acid-conjugated polyethylenimine (LA-PEI) and oxidized dextran. This material significantly reduced cytotoxicity and endowed the hydrogel with antioxidant properties [22]. Li et al. constructed a PEI-based double-network hydrogel that exhibited both the electrostatic antibacterial effect of PEI and the self-healing property of Schiff base crosslinking, enabling efficient repair of bacterially infected wounds [23]. Yang et al. further incorporated tobramycin into an OCS/PEI-based system for severe abdominal trauma treatment [24]. However, their work primarily focused on the therapeutic efficacy of drug-loaded formulations in acute trauma models. The OCS/PEI hydrogel system integrates the dynamic crosslinking property of oxidized CS with the adhesive and antibacterial functions of PEI, and may also offer a favorable physicochemical and biological microenvironment for wound healing. In this system, the oxidation degree of CS is a potentially adjustable parameter that could influence network structure and properties; however, this aspect has not been systematically explored.

In this study, we constructed a multifunctional oxidized chondroitin sulfate/polyethyleneimine (OCS/PEI, OPd) hydrogel through Schiff base crosslinking. Given the complementary properties of OCS and PEI, we hypothesized that their integration could generate a dynamically crosslinked hydrogel network with favorable physicochemical and biological characteristics for wound management. OPd hydrogels prepared using OCS with different oxidation degrees were systematically characterized for their microstructure, swelling behavior, mechanical and rheological properties, adhesive performance, antibacterial and antioxidant activities, and hemocompatibility and cytocompatibility. This study aimed to develop a multifunctional OCS/PEI hydrogel system that integrates injectability, tissue adhesion, mechanical stability, antibacterial and antioxidant activities, and biocompatibility for wound dressing applications.

## 2. Materials and Methods

### 2.1. Materials

Chondroitin sulfate (from pigs) (CS, 95%, Rhawn, Cat. No. R052165, Shanghai, China), sodium periodate (NaIO_4_, 99.5%, Macklin, Cat. No. S817518, Shanghai, China), and polyethyleneimine (PEI, M.W. = 18,000, Macklin, Cat. No. E960584, Shanghai, China) were purchased. Millipore water was prepared using a Milli-Q50 SP Reagent Water System (Millipore Corporation, Billerica, MA, USA). All purchased chemicals and solvents were used directly without further purification.

### 2.2. Preparation of OCS

We dissolved 4 g of CS in 50 mL of Millipore water and stirred it at room temperature until completely dissolved. Then, 3.9 g, 4.9 g, and 5.9 g of sodium periodate (NaIO_4_) were added to the solution, respectively, and the reaction was allowed to proceed in the dark for 24 h. Finally, the solution was dialyzed against deionized water using a dialysis bag with a molecular weight cut-off of 3500 Da, and the deionized water was replaced three times daily. After freeze-drying, samples with different degrees of oxidation were obtained and labeled as OCS1, OCS2, and OCS3 (Table 1).

### 2.3. Preparation of OCS/PEI Hydrogel

We dissolved 0.05 g of OCS in 0.75 mL of phosphate-buffered solution (PBS, pH 7.4) in a centrifuge tube and vortexed it until completely dissolved. Separately, 0.1 g of PEI was dissolved in 0.25 mL of PBS (pH 7.4) in a centrifuge tube and vortexed until completely dissolved. The two solutions were then mixed and vortexed rapidly to ensure thorough mixing and crosslinking, yielding OCS/PEI hydrogels. Three hydrogels were obtained, corresponding to different degrees of OCS oxidation, and were denoted as OPd1, OPd2, and OPd3 (Table 2).

### 2.4. Fourier Transform Infrared Spectroscopy (FT-IR)

The samples were characterized by Fourier transform infrared (FT-IR) spectroscopy using an attenuated total reflectance (ATR) accessory (Thermo Fisher, Nicolelis5, Waltham, MA, USA). FT-IR spectra with a resolution of 1 cm^−1^ were recorded in the range of 500–4000 cm^−1^. Each spectrum was scanned 64 times, and the average spectrum was obtained [25].

### 2.5. ^1^H Nuclear Magnetic Resonance (^1^H NMR) Spectroscopy

^1^H NMR spectra were recorded on a 600 MHz nuclear magnetic resonance spectrometer (JNM-ECZ600R/S3, JEOL, Tokyo, Japan), and the degree of grafting was determined accordingly. Briefly, 0.02 g of the sample was dissolved in 0.5 mL of heavy water (D_2_O) and transferred to an NMR tube for analysis.

### 2.6. Microscopic Structure Observation Using Scanning Electron Microscopy (SEM)

The OPd1, OPd2, and OPd3 hydrogel samples were frozen at −20 °C for 24 h, followed by freeze-drying for three days. The dried samples were cut into uniform blocks, mounted onto sample stages with conductive adhesive, and sputter-coated with platinum for 40 s to enhance the conductivity. Then, the morphology and structure were observed using a scanning electron microscope (SEM, Hitachi, Tokyo, Japan).

### 2.7. Swelling Performance Testing

The swelling ratios of the composite hydrogels were determined gravimetrically at room temperature. The freeze-dried hydrogel samples were weighed (Ms) and then fully immersed in PBS (pH 7.4). At predetermined time intervals (6, 12, 24, and 36 h), the samples were taken out, gently blotted with filter paper to remove surface water, and reweighed (Ma). The swelling ratio was calculated according to the following formula:

$$Swelling\;ratio\left(\%\right)=\frac{Ma-Ms}{Ms}\times 100\%$$

### 2.8. Rheological Performance Test

The rheological properties of the hydrogels were tested using a TA rheometer (DHR-2, New Castle, DE, USA) at 37 °C with a constant strain of 1% and a constant frequency of 10 rad/s [26]. The storage modulus (G′) and loss modulus (G″) were measured using an aluminum plate (20 mm diameter) sealed with silicone oil. The linear viscoelastic region was determined by oscillatory strain sweeps from 0.1% to 500% at a fixed angular frequency of 10 rad/s. Frequency sweep tests were then performed within the range of 0–10 rad/s, under a constant strain (γ) of 0.5% within the linear viscoelastic region.

### 2.9. Self-Healing Performance

A hydrogel sample was cut into two equal halves, one of which was stained with methylene blue solution. The two halves were then brought into close contact. The self-repairing behavior was observed and recorded at predetermined time intervals.

The hydrogel was placed on the plate of a TA rheometer, and the edges were sealed with silicone oil to prevent water evaporation. Amplitude sweep tests were conducted at 25 °C with a fixed angular frequency of 10 rad/s over a strain range of 0.1% to 600% to identify the critical strain region. To evaluate self-healing ability, the strain was alternated between 1.0% (small strain) and 400% (large strain), with each strain stage lasting 100 s.

### 2.10. Adhesion Performance Test

The adhesion performance of the hydrogels was evaluated on different materials, including wood, glass, plastic, metal, and rubber. Their adhesion to various organs (heart, liver, spleen, lung, and stomach) was also examined. The adhesion was documented by photographic images. The animal study protocol was approved by the Ethics Committee of Xinyang Normal University (XFEC-2026-074; date of approval: 2 January 2026).

### 2.11. Injectability Test

The injectability of the hydrogel was measured using a TA rheometer (DHR-2, New Castle, DE, USA) at 25 °C. A constant strain of 1% was applied, and the shear rate varied from 0.1 rad/s to 100 rad/s. Twenty consecutive oscillatory frequency sweep experiments were conducted. In addition, the prepared hydrogel was loaded into a syringe and extruded to write letters such as “OPd1”.

### 2.12. Compression Performance Test

First, the hydrogel was prepared into cylindrical specimens of uniform dimensions, with a cross-sectional area of approximately 121 mm^2^ for compression. Uniaxial compression tests were performed using a universal material testing machine (MT5700, Suzhou Yongqinquan Intelligent Equipment Co., Ltd., Suzhou, China) at room temperature at 10 mm/min, and each formulation was tested in triplicate. Cyclic compression tests were then conducted on each hydrogel formulation to evaluate the material’s resilience and fatigue resistance.

### 2.13. Antibacterial Performance Testing

The optical density of the bacterial suspension at 600 nm (OD_600_) was measured using a Spectra Max 190 microplate reader (Molecular Devices, Berlin, Germany) and adjusted to approximately 0.6–0.8. Then, 0.02 g of freeze-dried hydrogel was added to 10 mL of LB medium containing 10 μL of bacterial suspension (*E. coli* or *S. aureus*). The mixed solution was incubated in a shaker at 37 °C and 180 rpm for 6 h. After incubation, the OD_600_ values were measured. Three replicates were performed for each sample, and the antibacterial rate was calculated according to the following formula:

$$Antibacterial\;Rate\left(\%\right)=\frac{{OD}_{control}-{OD}_{sample}}{{OD}_{control}}\times 100\%$$

where OD_control_ and OD_sample_ are the absorption values of the blank control group and the sample group, respectively.

### 2.14. Oxidation Resistance Performance Test

The antioxidant activity of the OPd hydrogels was investigated using the 2,2′-azino-bis (3-ethylbenzothiazoline-6-sulfonic acid) diammonium salt (ABTS) radical scavenging assay. The ABTS radical cation (ABTS•+) stock solution (Macklin, Cat. No. A800764) was generated by reacting ABTS (12 mg in 3 mL of water) with potassium persulfate (2 mg in 3 mL of water) in the dark at 4 °C for 12 h. The stock solution was diluted with deionized water to obtain an 8% ABTS working solution. Subsequently, 0.03 g of the freeze-dried hydrogel sample was added to 5 mL of the working solution in the dark, mixed thoroughly, and allowed to stand. The absorbance of the mixture was measured at 734 nm using a microplate reader (EnSpire, PerkinElmer, Waltham, MA, USA). The ABTS scavenging activity was calculated according to the following formula:

$$ABTS\;Scavenging\;Activity\left(\%\right)=\frac{Ac-Ab}{Ac}\times 100\%$$

where Ac is the absorbance of the blank group, and Ab is the absorbance of the sample group.

### 2.15. In Vitro Hemolysis Test

Red blood cells were isolated from sterile anticoagulated sheep whole blood (containing EDTA) (BKMALAB, Cat. No. 110805018, Hunan, China) by centrifugation. Cells were washed with saline and centrifuged repeatedly until the supernatant was clear, yielding a red blood cell suspension. Then, 2.5 mg of the hydrogel was added to 1 mL of normal saline and incubated for 30 min. After that, 20 μL of the red blood cell suspension was added, and the mixture was incubated at 37 °C for 1 h. For the positive control, 1 mL of deionized water was mixed with 20 μL of the red blood cell suspension and incubated for 1 h. For the negative control group, 1 mL of normal saline was mixed with 20 μL of red blood cell suspension and incubated for 1 h. Finally, the hemolysis rate of the sample was calculated according to the following formula:

$$Hemolysis\;Rate\left(\%\right)=\frac{As-An}{Ap-An}\times 100$$

where As, Ap, and An are the absorbance values corresponding to the sample group, the positive control group, and the negative control group, respectively.

### 2.16. In Vitro Cell Compatibility Test

Prior to the experiment, hydrogel extracts were prepared as follows: the test samples were pretreated by immersion in 75% (volume fraction) ethanol and UV irradiation for 12 h, and then repeatedly rinsed with sterile PBS at least three times to remove residual ethanol. Subsequently, an equal volume of complete medium (formulated with 10% fetal bovine serum and 1% penicillin–streptomycin in DMEM medium) was added to each sample. The mixture was incubated at 37 °C for 24 h to obtain the hydrogel extract. For the CCK-8 assay (APExBIO, Cat. No. K1018, Houston, TX, USA), NIH-3T3 cells (ATCCCRL-1658TM, Manassas, VA, USA) were seeded into a 96-well plate at approximately 5000 cells/well. Afterward, the cells were cultured with the hydrogel extract in a humidified incubator at 37 °C with 5% CO_2_. At predetermined time points (days 1 and 2), 10% (*v*/*v*) CCK-8 solution was added to measure the absorbance at 450 nm. Cell viability was calculated using the following formula:

$$Cell\;viability\left(\%\right)=\frac{As-Ab}{Ac-Ab}\times 100$$

where As, Ac, and Ab refer to the absorbance values from the sample group, the control group, and the blank group, respectively.

### 2.17. In Vitro Cellular ROS Scavenging Assay

The cellular reactive oxygen species (ROS) scavenging activity of the hydrogel was measured using an ROS detection kit (DCFH-DA, Beyotime, Shanghai, China). Cells were seeded at 300,000 cells per well and cultured for 24 h. Then, an H_2_O_2_ working solution was prepared. The culture medium was removed, and the cells were washed twice with PBS. The cells were divided into three groups: the blank group (cells cultured with complete medium), the positive control group (cells treated with H_2_O_2_ working solution), and the sample group (cells treated with H_2_O_2_ working solution and hydrogel extract). Following 2 h of dark incubation, cells were stained with DCFH-DA for 30 min, washed with PBS, and visualized by fluorescence microscopy.

### 2.18. Live and Dead Cell Staining

NIH-3T3 cells (500,000 cells) were seeded in 35 mm diameter cell culture dishes and co-cultured with the composite hydrogel extract. The culture was terminated on days 1, 2, and 3, followed by staining using a Live/Dead Cell Staining Kit (Beyotime, Cat. No. C20151, Shanghai, China). The stained samples were visualized under a confocal laser scanning microscope: dead cells appeared red, while live cells appeared green.

### 2.19. Cell Migration Assay

A cell-scratch plug-in device (ibidi 2-well Culture insert, ibidi GmbH, Gräfelfing, Germany) was placed into a 6-well plate using tweezers. Then, 50,000 NIH-3T3 cells were seeded into each well of the insert. The cells were incubated for 24 h. After the cells reached full confluence, the insert was gently removed. After washing with PBS, the experimental group was cultured with hydrogel extract-containing medium supplemented with 1% fetal bovine serum (FBS). The control group was cultured with medium alone under the same conditions. The cells were further incubated, and images were captured at 6 h and 12 h post-treatment.

### 2.20. Statistical Analysis

All results were expressed as mean ± standard deviation (SD). Statistical evaluations were performed using SPSS 27.0 and GraphPad Prism 10.0. One-way ANOVA was performed using SPSS for the swelling, antibacterial, and ABTS assay data, and using GraphPad Prism for the hemolysis and ROS fluorescence measurements. Two-way ANOVA (GraphPad Prism) was employed for CCK-8 cell viability. Following ANOVA, all post hoc pairwise comparisons were conducted with Tukey’s multiple comparisons test. A *p*-value < 0.05 was considered statistically significant, with significance levels designated as * *p* < 0.05, ** *p* < 0.01, and *** *p* < 0.001.

## 3. Result and Discussion

### 3.1. Fabrication of OCS Hydrogels

**Figure 1:**
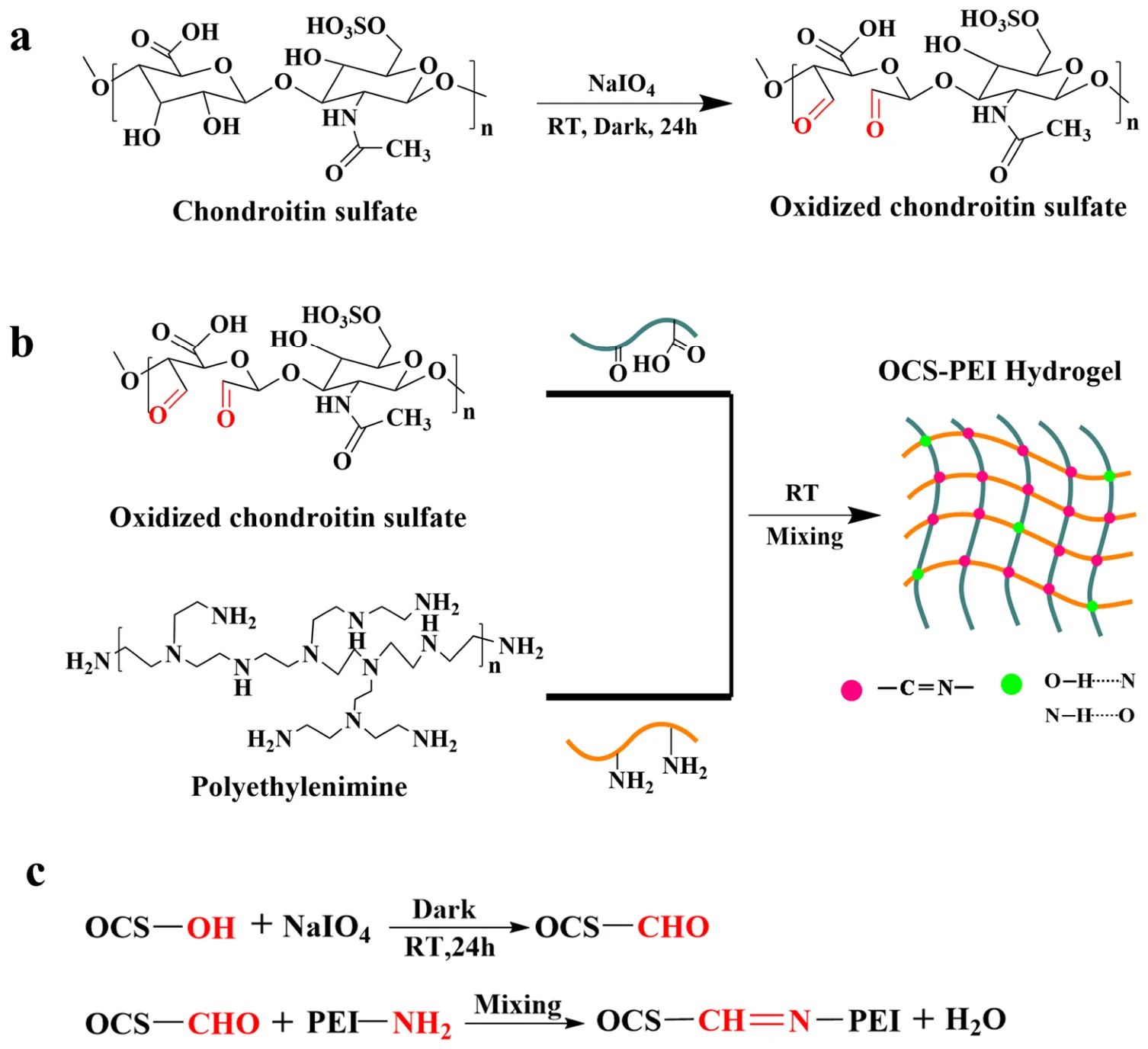
Preparation and gelation process of OCS/PEI hydrogels. (**a**) Chemical reaction route for the preparation of OCS via NaIO_4_ oxidation of CS. (**b**) Schematic diagram of the crosslinking of OCS with PEI to form OCS/PEI hydrogels, with Schiff base bonds (-C=N-) and hydrogen bonds. (**c**) Simplified chemical equations showing the two-step reaction: oxidation of chondroitin sulfate and subsequent Schiff-base condensation between aldehyde groups of OCS and amino groups of PEI.

The chemical synthesis route and structural characterization of OCS and OCS/PEI hydrogels are illustrated in Figure 1. As shown in Figure 1a, active aldehyde groups were introduced into the sugar rings of chondroitin sulfate (CS) via NaIO_4_ oxidation. Different amounts of NaIO_4_ (3.9 g, 4.9 g, and 5.9 g) were deliberately used to obtain OCS with varying oxidation degrees (OCS1, OCS2, and OCS3, respectively). Subsequently, the aldehyde-rich OCS was mixed with PEI. A three-dimensional crosslinked network was constructed through Schiff base reactions between aldehyde and amino groups, forming dynamic covalent bonds (-C=N-) (Figure 1b).

**Figure 2:**
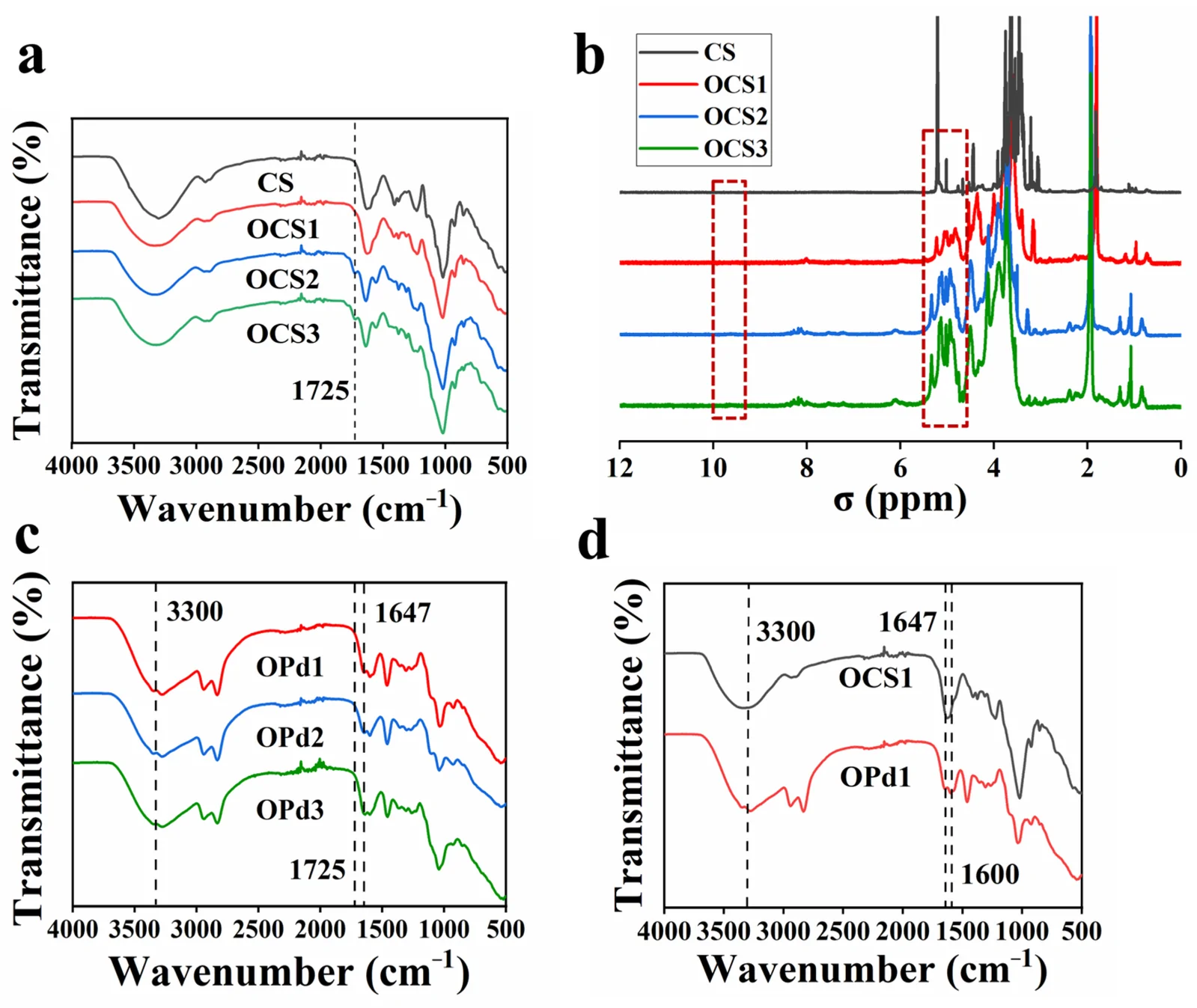
Spectral analysis of precursors and crosslinked networks. (**a**) FT-IR spectra of CS, OCS1, OCS2, OCS3. (**b**) ^1^H NMR spectra of CS, OCS1, OCS2, and OCS3. (**c**) FT-IR spectra of OCS/PEI hydrogels (OPd1, OPd2, OPd3). (**d**) Comparative FT-IR spectra of OCS1 and its corresponding hydrogel OPd1.

To confirm the chemical modifications and crosslinking reactions, FT-IR spectroscopy was employed because it provides direct evidence of functional group transformations through characteristic vibrational bands. As displayed in Figure 2a, a distinct new absorption peak centered at 1725 cm^−1^ was observed for the oxidized products (OCS1, OCS2, and OCS3) compared with unmodified CS. This peak was assigned to the carbonyl (C=O) stretching vibration of the aldehyde groups, confirming the successful oxidation of the CS backbone. Notably, peak intensity increased with higher NaIO_4_ concentrations (OCS1 < OCS2 < OCS3), indicating that aldehyde content is tunable by adjusting the oxidation degree, a critical design feature for controlling subsequent crosslinking density and hydrogel properties. ^1^H NMR spectroscopy was performed to verify the structural changes (Figure 2b). Furthermore, spectral variations were observed in the sugar ring proton region (4.0–6.0 ppm) for the oxidized products (OCS1, OCS2, and OCS3). In contrast, the acetyl methyl proton signals in the 1.8–2.2 ppm region remained unchanged, confirming that the oxidation specifically targeted the diol groups without affecting the acetamide side chains. The characteristic signal of aldehyde protons in the 9–11 ppm region was relatively weak. This is mainly because the aldehyde groups in the polysaccharide system are prone to hydration to form hemiacetal structures, which induces dynamic proton exchange and thus leads to a certain degree of signal attenuation [27].

Following crosslinking with PEI, notable spectral changes were observed in the OPd hydrogels (Figure 2c). The aldehyde peak at 1725 cm^−1^ was markedly attenuated or nearly disappeared, indicating the consumption of aldehyde groups during the Schiff base reaction. Meanwhile, the broad absorption band at 3300 cm^−1^ was altered due to the overlapping stretching vibrations of hydroxyl (-OH) and amino (-NH_2_) groups. Most notably, a new characteristic peak emerged at 1647 cm^−1^, which was attributed to the C=N stretching vibration of the newly formed imine bonds. Collectively, the disappearance of the aldehyde peak and the emergence of the C=N stretching band provided direct spectroscopic evidence for the successful construction of the Schiff base crosslinked network. In addition, the peak near 1600 cm^−1^, which is assigned to the N-H bending vibration of amino groups in PEI, shows a significant change in peak shape, proving that the amino groups on PEI have been consumed by participating in the Schiff base reaction. These structural changes confirm that oxidation endowed CS with aldehyde functionalities and that these groups subsequently participated in dynamic Schiff base crosslinking with PEI, providing the molecular basis for the formation of the OPd hydrogel network.

### 3.2. Microstructure and Water Absorption Properties of OPd Hydrogels

**Figure 3:**
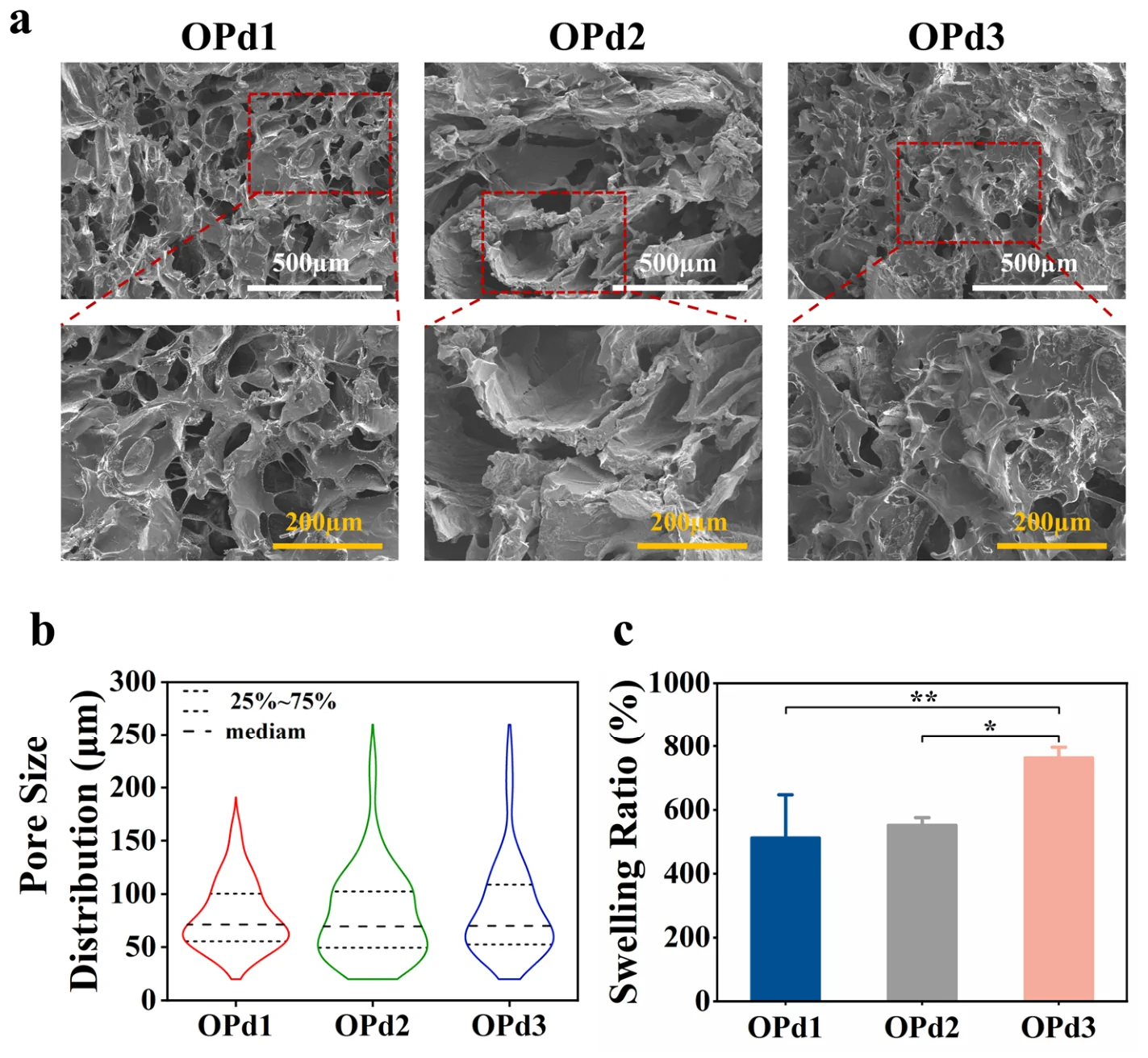
Microstructure and swelling properties of OCS/PEI hydrogels (OPd1-OPd3). (**a**) SEM images of OPd1, OPd2, and OPd3 hydrogels. (**b**) Pore size distribution of OPd1, OPd2, and OPd3 hydrogels. (**c**) Comparative histogram of equilibrium swelling rates of OPd1, OPd2, and OPd3 hydrogels (*, *p* < 0.05; **, *p* < 0.01).

The microscopic morphology and swelling behavior of the hydrogels are shown in Figure 3. The SEM images (Figure 3a) revealed that all OPd hydrogels possessed highly interconnected three-dimensional porous network structures. The pore sizes of the hydrogels were mainly distributed between 50 and 250 μm (Figure 3b). This pore size range is notable because it falls within the optimal window (50–300 μm) reported for tissue engineering scaffolds, which facilitates cell adhesion, migration, and vascularization while maintaining sufficient mechanical integrity [28]. As shown in the statistical analysis in Figure 3c, the equilibrium swelling ratio of OPd3 was significantly higher than those of OPd1 and OPd2. The underlying mechanism is that although a higher oxidation degree introduced more reactive aldehyde groups, the oxidation of vicinal diols by NaIO_4_ inevitably caused considerable degradation of the polysaccharide backbone and a substantial reduction in molecular weight. Consequently, the effective crosslinking density was substantially reduced during subsequent gelation, leading to a looser three-dimensional network and larger pores, which ultimately facilitated water permeation and swelling. Therefore, the increased swelling of OPd3 suggests that the oxidation degree can influence the hydration behavior of the hydrogel network, although the present results do not establish a direct quantitative relationship between oxidation degree and crosslinking density.

The favorable interconnected porous structure and high swelling capacity endowed the hydrogels with practical application advantages. Firstly, the macroporous network simulated the three-dimensional environment of the native extracellular matrix (ECM), providing ample space for cell adhesion, proliferation, and migration. Meanwhile, the high porosity facilitated efficient diffusion of oxygen and nutrients into the hydrogel interior while allowing for the timely removal of metabolic waste. This feature is crucial for their role as ideal candidate materials for tissue engineering scaffolds [29]. Secondly, the high water uptake and swelling capability made these hydrogels highly attractive for wound repair applications. In wound dressing applications, the high swelling ratio contributed to effective absorption of wound exudates, maintained a moist local environment conducive to wet healing, and prevented wound dehydration and secondary infections, thereby accelerating the skin healing process [30,31,32]. Thus, the porous and highly hydrated structure observed here provides a favorable physical basis for maintaining a moist wound environment and managing wound exudates.

### 3.3. Rheological Behavior and Self-Healing Ability of OPd Hydrogels

**Figure 4:**
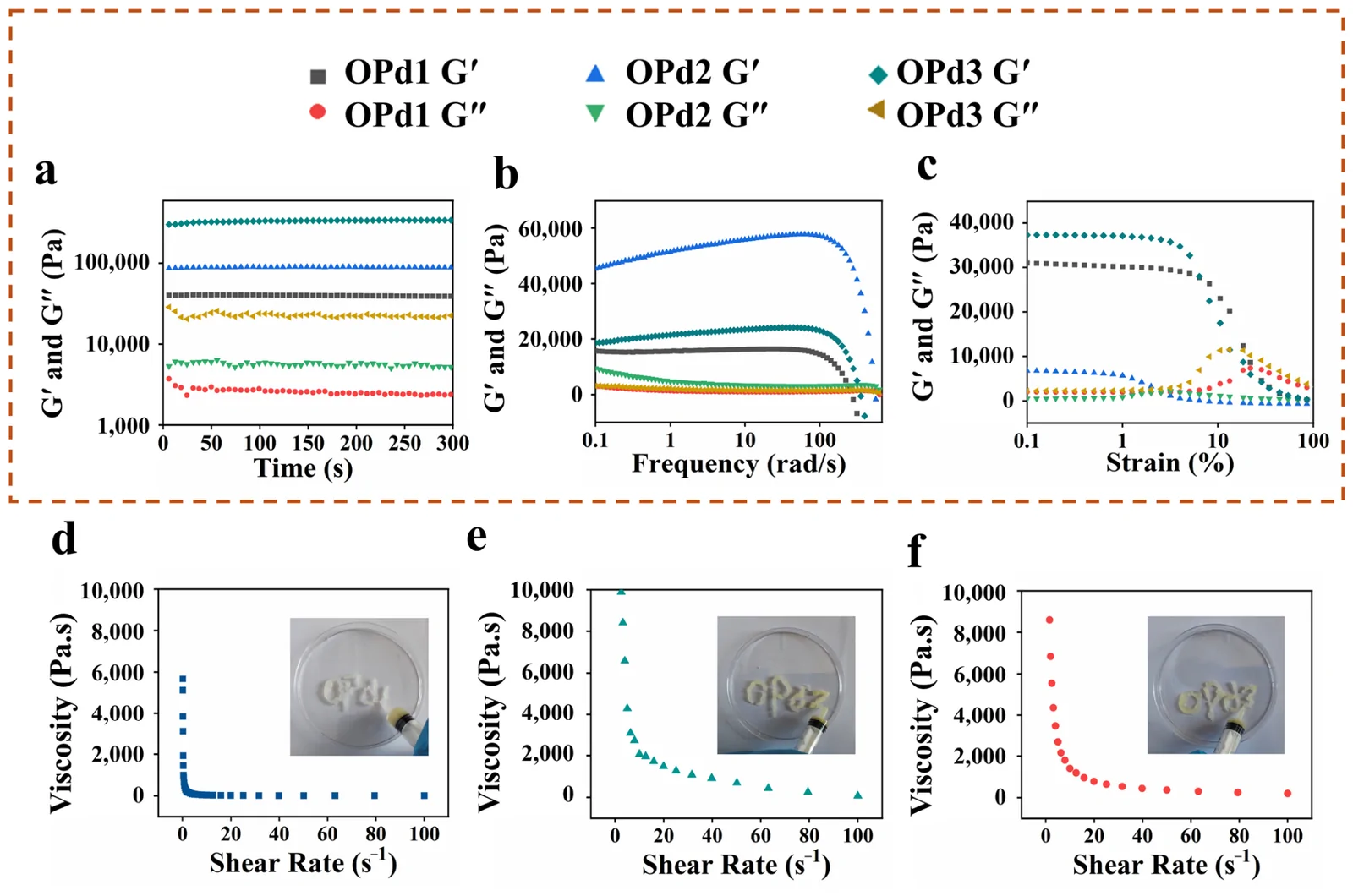
Rheological evaluation and shear-dependent viscosity of hydrogels. Storage modulus (G′) and loss modulus (G″) as functions of time (**a**), frequency (**b**), and strain (**c**) for OPd1, OPd2, and OPd3 hydrogels. Viscosity versus shear rate curves of OPd1 (**d**), OPd2 (**e**), and OPd3 (**f**) hydrogels at room temperature (25 °C), demonstrating their shear-thinning injectability.

Rheological characterization was performed to determine whether the OPd hydrogels could maintain a stable viscoelastic network while undergoing deformation, and to evaluate their suitability for injectable wound dressing applications. Rheological characterization of the storage modulus (G′) and loss modulus (G″) provides valuable insights into the biomedical potential of hydrogels, including their capacity to modulate the viscoelastic behavior and mechanical stability of biological tissues [33,34]. Rheological measurements were performed to evaluate the viscoelastic properties of OPd1, OPd2, and OPd3 hydrogels (Figure 4a–c). In the time (Figure 4a) and frequency (Figure 4b) sweep tests, the storage modulus (G′) of all samples was higher than the loss modulus (G″). Among the three groups, OPd2 exhibited the highest G′, followed by OPd3, while OPd1 showed the lowest value (Figure 4b). In the strain sweep test, the modulus decreased gradually with increasing strain amplitude (Figure 4c). This decrease indicates progressive disruption of the network under increasing deformation and provides a basis for evaluating the ability of the dynamic network to undergo structural rearrangement.

The shear-thinning behavior and injectability of the hydrogels were further confirmed at room temperature (Figure 4d–f). The viscosity–shear rate relationship was examined because shear-thinning behavior is particularly important for injectable hydrogels: a decrease in viscosity under shear facilitates extrusion through a needle, whereas recovery of the network after injection helps the material retain its structural integrity at the application site. In the viscosity–shear rate curves, a high initial viscosity was exhibited by all samples at low shear rates, followed by a sharp drop as the shear rate increased. This rheological property endowed the hydrogels with favorable injectability, as directly confirmed by optical images. These images showed the extrusion of the gels through needles while maintaining specific letter shapes.

**Figure 5:**
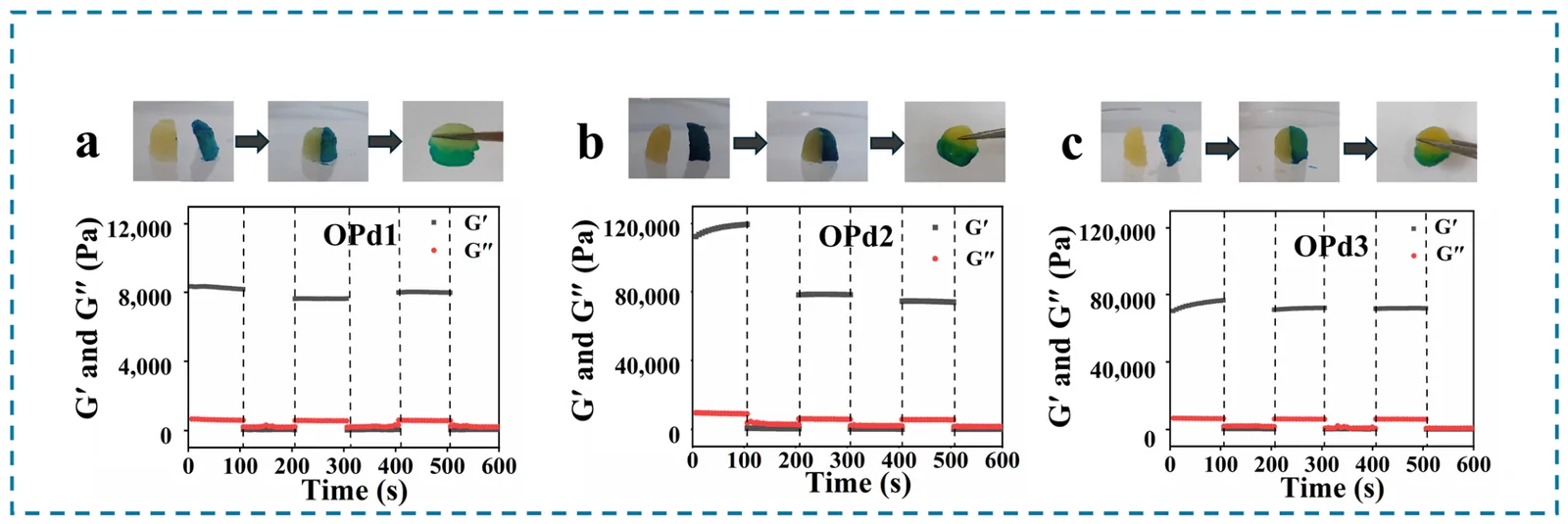
The self-healing process and the corresponding step-strain cyclic tests (variations in storage modulus G′ and loss modulus G″ as a function of time) of OPd1 (**a**), OPd2 (**b**), and OPd3 (**c**) hydrogels.

Furthermore, the self-healing behavior of the OPd hydrogels was characterized by step-strain cyclic measurements and visual inspection (Figure 5a–c). This test was used to determine whether the dynamic network could rapidly recover after mechanical disruption, an important characteristic for dressings exposed to repeated deformation during body movement. At high strain amplitude, the network structure was disrupted, resulting in a loss modulus greater than the storage modulus. Upon rapid strain recovery, both the storage and loss moduli returned to their original levels. Notably, this reversible gel–sol transition remained reproducible throughout repeated cycles. This self-healing property was mainly attributed to the reversible breakage and reformation of dynamic Schiff base (imine) bonds and hydrogen bonds within the network. Rapid re-crosslinking occurred between free aldehyde and amino groups, along with reconstruction of physical interactions. These processes collectively enabled quick recovery of the gel structure and mechanical performance [35,36,37]. The repeated recovery observed in the cyclic strain test therefore supports the proposed role of dynamic covalent and non-covalent interactions in maintaining the structural integrity of the OPd hydrogels after mechanical damage.

### 3.4. Adhesion Performance and Compressive Mechanical Properties of OPd Hydrogels

**Figure 6:**
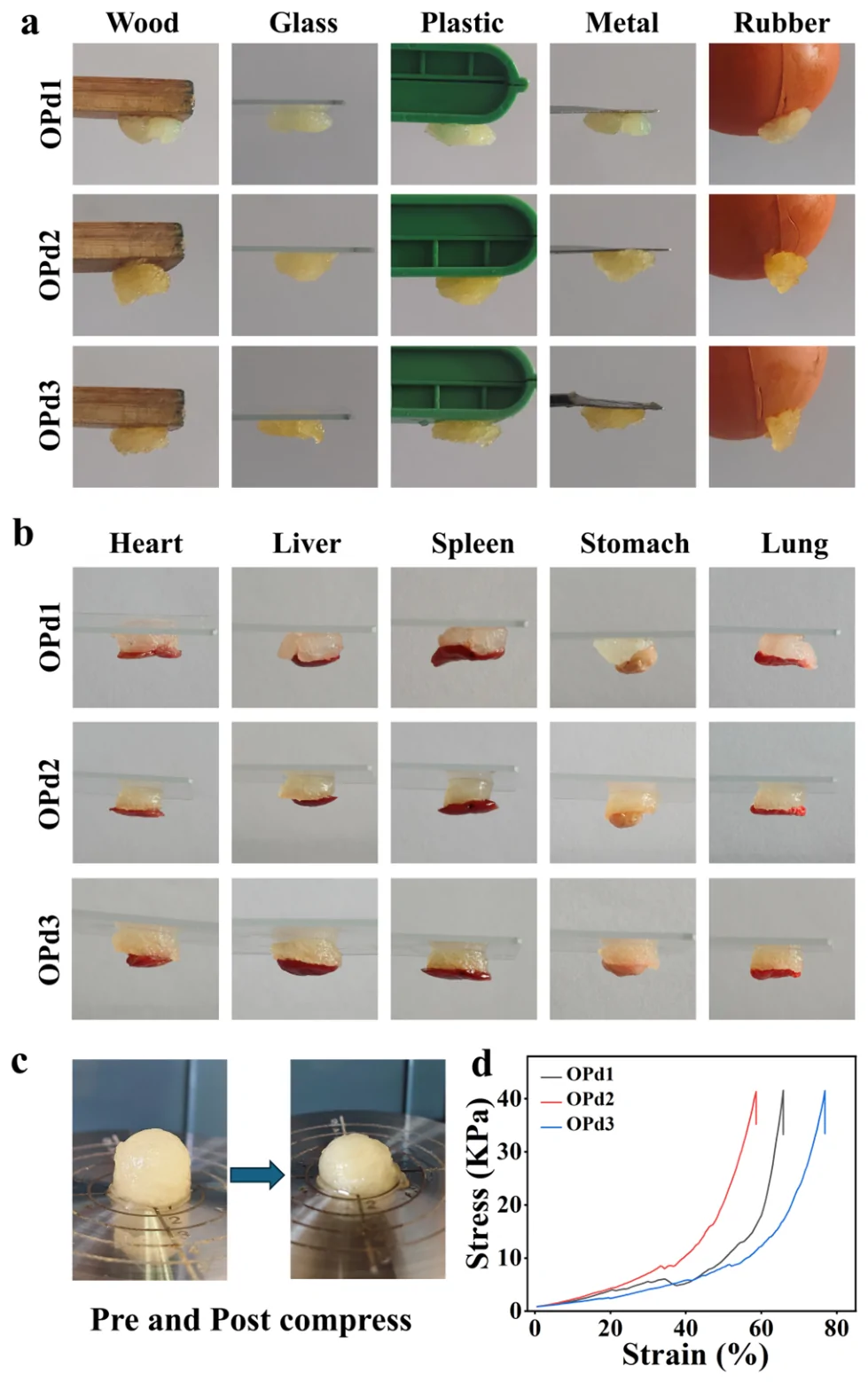
Adhesive and compressive behavior of OPd hydrogels. (**a**) Adhesion effects of OPd hydrogels on different abiotic materials (wood, glass, plastic, metal, and rubber). (**b**) Adhesion effects of OPd hydrogels on various biological tissues (heart, liver, spleen, stomach, and lung). (**c**) Optical photographs of the hydrogels before and after compression. (**d**) Compressive stress–strain curves of the hydrogels.

Because a wound dressing must remain in close contact with irregular and dynamically moving tissue surfaces, adhesion and mechanical stability were further evaluated to determine whether the OPd hydrogels could maintain interfacial contact under physiological conditions. The adhesive properties were evaluated by adhering them to the surfaces of various materials. OPd hydrogels exhibited strong adhesion to various non-biological materials (wood, glass, plastic, metal, and rubber) (Figure 6a) and biological tissues (heart, liver, spleen, stomach, and lung) (Figure 6b). The OPd hydrogels firmly adhered to all tested surfaces, remaining stable under hanging conditions. This broad-spectrum adhesion originates from a synergistic mechanism. The abundant aldehyde and amino functionalities within the OPd network provide multiple possibilities for interfacial interactions. On the one hand, the unreacted aldehyde groups in the hydrogel network form covalent Schiff base bonds with amino groups on the substrate surfaces. On the other hand, the abundant amino groups on the PEI chains endow the hydrogels with strong positive charges, enabling powerful non-covalent interactions—including electrostatic attraction and hydrogen bonding—with negatively charged or polar surfaces [38]. The coexistence of covalent and non-covalent interactions may therefore account for the broad adhesion observed across substrates with different surface chemistries. The synergistic effect of covalent and non-covalent interactions imparts the hydrogels with universal adhesion to diverse substrates. In practical applications, this adhesion property is advantageous. For instance, as a tissue sealant, the hydrogel can rapidly adhere to and seal surgical incisions or organ wounds, preventing postoperative exudation and bleeding [39]. As a wound dressing, it can intimately attach to irregular wound surfaces while maintaining interfacial adhesion during tissue movement, providing a physical barrier conducive to wound healing [40,41].

Furthermore, after high-intensity compression, hydrogels maintained their structural integrity (Figure 6c). The smooth stress–strain curves further indicated their robust compressive properties and structural stability (Figure 6d). The compressive response reflects the ability of the interconnected polymer network to distribute and withstand external loads without catastrophic structural failure. This elastic recovery was mainly attributed to the stable three-dimensional network formed by Schiff base crosslinking, which effectively absorbed mechanical stress. Together with the self-healing behavior observed above, these results suggest that the dynamically crosslinked network can accommodate mechanical deformation while retaining overall structural integrity. These mechanical properties enabled the hydrogels to maintain their function in dynamic physiological environments when used as compressible implants or tissue dressings.

### 3.5. Antibacterial, Antioxidant, and Hemocompatibility Evaluation of OPd Hydrogels

**Figure 7:**
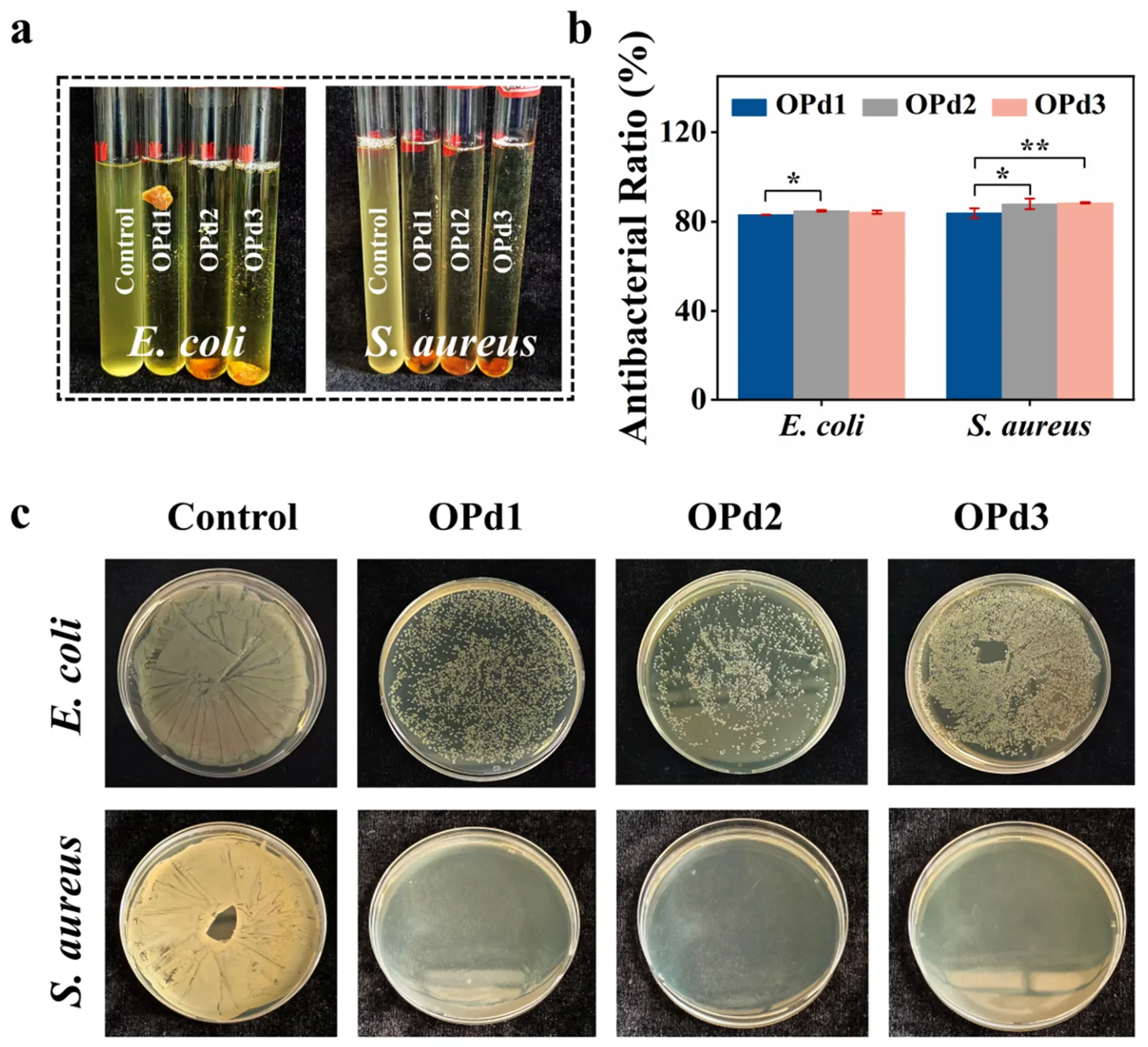
Antibacterial activity of OPd hydrogels. (**a**) Representative images of the hydrogels against *E. coli* and *S. aureus* in LB liquid medium. (**b**) The antibacterial ratios of hydrogels against *E. coli* and *S. aureus*. (**c**) Representative images display the hydrogels on a nutrient agar plate against *E. coli* and *S. aureus*. *, *p* < 0.05; **, *p* < 0.01.

Antibacterial activity was evaluated against both Gram-negative (*E. coli*) and Gram-positive *S. aureus* (*S. aureus*) to determine whether the OPd hydrogels could provide broad-spectrum protection against bacterial contamination, which is particularly relevant to wound dressings. A marked decrease in the turbidity of both bacterial cultures was observed upon incubation with the hydrogels (Figure 7a). Quantitatively, the inhibition rates against *E. coli* and *S. aureus* exceeded 80% (Figure 7b), demonstrating the potent antibacterial efficacy of the hydrogels. In addition, the visible reduction in colony numbers for the hydrogel-treated groups relative to the untreated control (Figure 7c) served as further confirmation of the potent antibacterial efficacy of the hydrogels [26]. The outstanding antibacterial performance of the hydrogels can be attributed to the synergistic effect of PEI-mediated membrane disruption and OCS-induced oxidative stress. On the one hand, PEI, rich in protonated amino groups, endows the hydrogel surface with abundant positive charges, enabling electrostatic capture of negatively charged bacteria. This is followed by membrane disruption and subsequent cytoplasmic leakage. On the other hand, OCS triggers excessive intracellular reactive oxygen species (ROS) accumulation, resulting in oxidative damage. The consistent inhibition of both Gram-negative and Gram-positive bacteria indicates that the OPd hydrogels possess broad-spectrum antibacterial activity, which is desirable for reducing the risk of bacterial colonization in wound environments.

**Figure 8:**
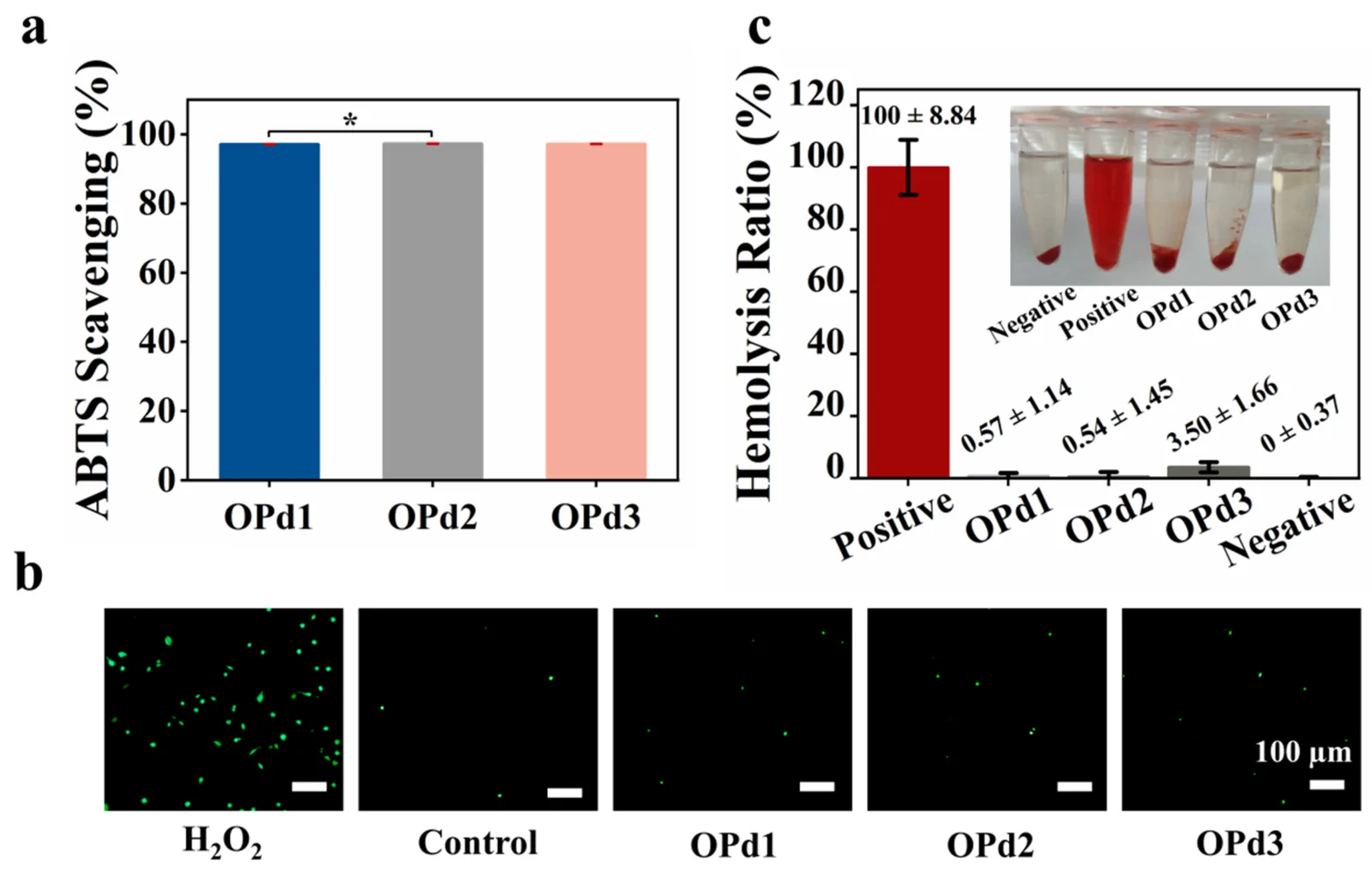
Antioxidant capacity and hemocompatibility of OPd hydrogels. (**a**) ABTS free radical scavenging rate of hydrogels. (**b**) DCFH-DA assay of intracellular ROS-scavenging activity of hydrogels. (**c**) Hemolysis evaluation of hydrogels. *, *p* < 0.05.

Scavenging excessive ROS and restoring redox homeostasis in infected wounds are critical indicators for evaluating the pro-healing potential of novel antimicrobial dressings. An ABTS assay was used to evaluate the antioxidant activity and preliminary wound oxidative stress-relieving potential of the samples. All hydrogel treatment groups achieved ABTS scavenging rates exceeding 90% (Figure 8a), indicating that each group possessed remarkably potent and consistent free-radical-scavenging activity. This consistently high scavenging efficiency suggests that the OPd hydrogel network possesses substantial intrinsic radical-scavenging capacity. The abundant amino groups of PEI may contribute to radical interactions through electron- or hydrogen-donating processes, although the relative contribution of individual components was not separately determined in this study. Moreover, we employed the DCFH-DA staining assay to measure intracellular reactive oxygen species levels in hydrogel-treated cells [42]. This cellular assay was further used to determine whether the antioxidant activity observed in the chemical assay could translate into regulation of intracellular oxidative stress. As shown in Figure 8b, H_2_O_2_ stimulation caused intense green fluorescence in the positive control group. Notably, this signal was markedly attenuated in the hydrogel-treated groups, indicating a reduction in intracellular ROS. The reduction in intracellular ROS, together with the strong ABTS scavenging activity, suggests that the OPd hydrogels may help maintain a more favorable redox microenvironment during wound repair.

To evaluate the blood compatibility of the hydrogels for wound dressing applications, a hemolysis test was conducted. Hemolysis testing was selected because direct contact between a wound dressing and blood can cause erythrocyte membrane damage, making blood compatibility an important prerequisite for wound-contacting biomaterials. The hemolysis ratios of all hydrogel-treated groups were below 5%, meeting the acceptable safety threshold (Figure 8c). The hemolysis ratio demonstrated a clear oxidation degree-dependent increase, with the highest oxidation group (OPd3) approaching the 5% safety threshold. These results indicate that the hydrogel formulations with OPd2 and lower oxidation degrees possess good erythrocyte compatibility. However, further oxidation to OPd3 considerably increased the risk of erythrocyte membrane damage.

### 3.6. Cytocompatibility of OPd Hydrogels

**Figure 9:**
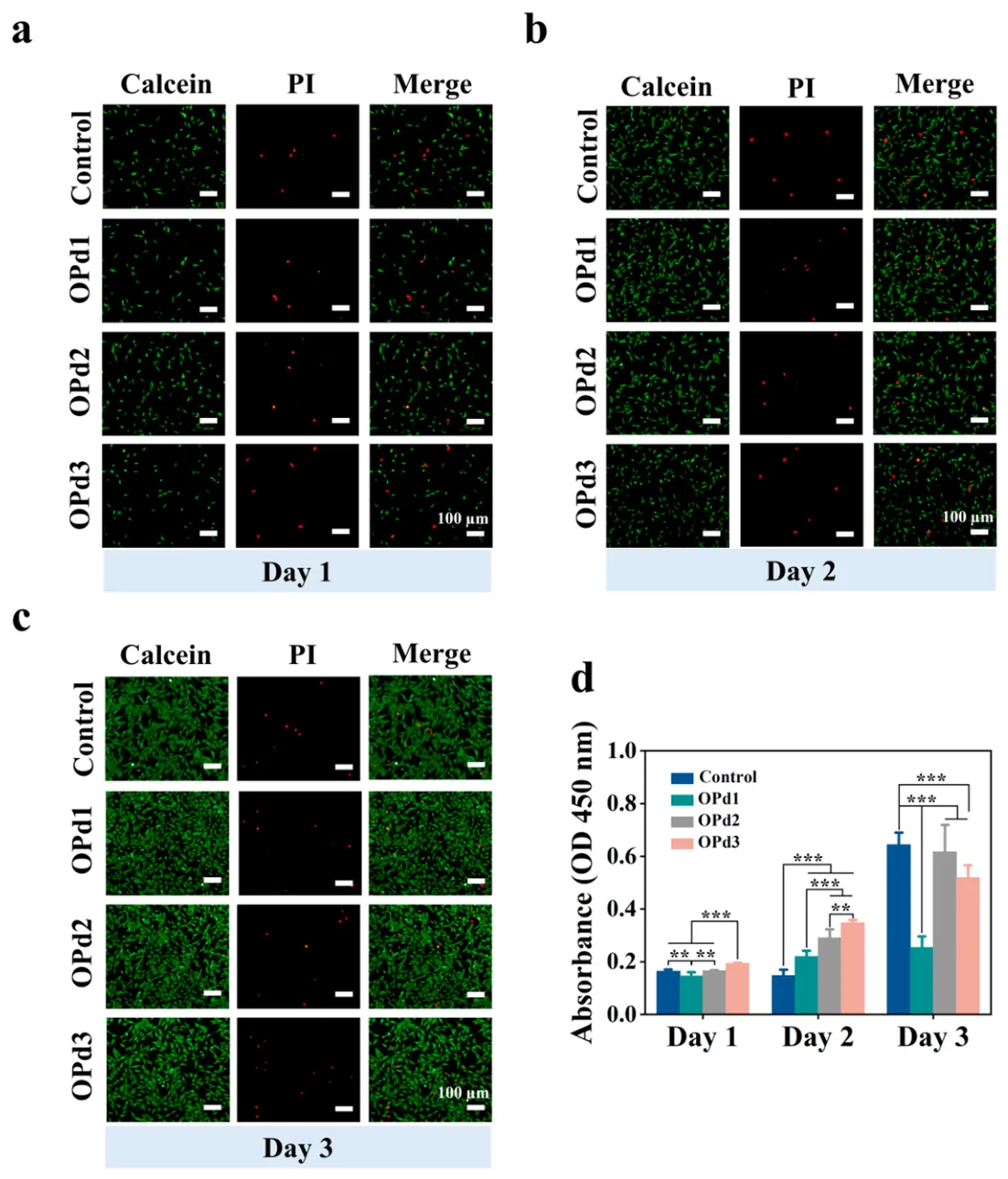
In vitro cytotoxicity and proliferation assay of OPd hydrogels. Cell viability of NIH 3T3 cells exposed to hydrogel extracts was assessed over 1 (**a**), 2 (**b**), and 3 (**c**) days using Calcein-AM (live cells, green) and propidium iodide (dead cells, red) dual fluorescence staining. Scale bar: 100 μm. (**d**) CCK-8 cell proliferation assay results of each group at Day 1, 2, and 3, determined by OD 450 nm absorbance. **, *p* < 0.01, and ***, *p* < 0.001.

Cytocompatibility was subsequently evaluated because a wound dressing should not only provide physical and antibacterial functions but also maintain a cellular environment compatible with tissue regeneration. To assess cell viability, Calcein-AM/PI double staining was applied. As presented in Figure 9a–c, abundant green fluorescence and very few red fluorescent dead cells were observed in the control group and all OPd treatment groups (OPd1–OPd3). No marked increase in dead cell count was detected across any group, and the cells consistently maintained a well-spread, healthy morphology. These qualitative observations indicate that direct exposure to the OPd hydrogels did not cause obvious cytotoxic effects. To further quantitatively evaluate cell viability and proliferation, CCK-8 assays were performed. During the 3-day culture period, absorbance at 450 nm increased gradually in all groups (Figure 9d). Although the control group showed the highest absorbance on Day 3, the OPd1, OPd2, and OPd3 hydrogel groups also maintained steady growth. These data collectively indicated that the hydrogels exhibited favorable cytocompatibility and effectively supported cell proliferation. The comparable increase in metabolic activity over time among the hydrogel groups further suggests that the OPd formulations provided a cell-compatible microenvironment without markedly impairing cell proliferation.

**Figure 10:**
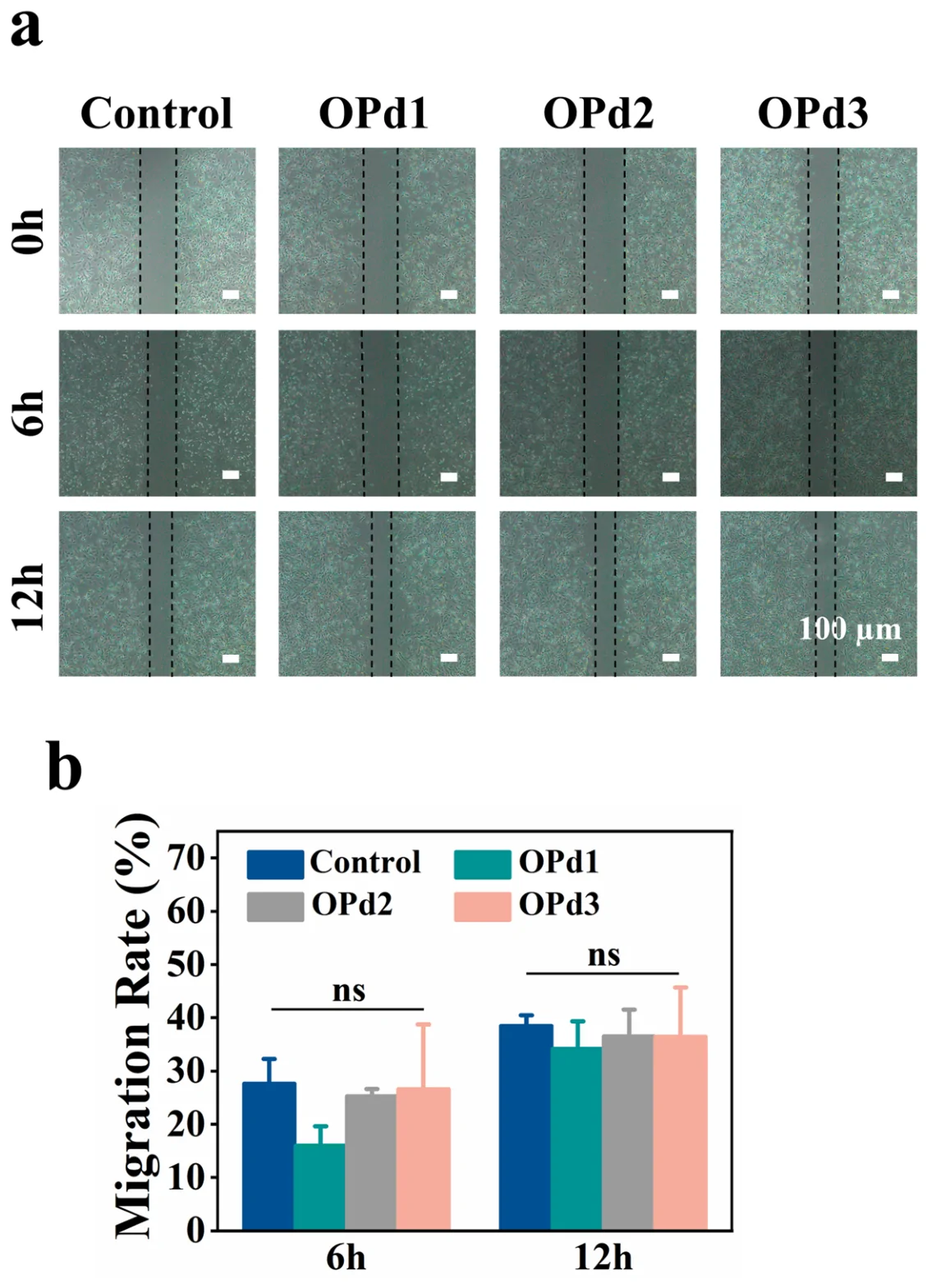
Cell migration promotion by OPd hydrogels in vitro. (**a**) Representative images of NIH 3T3 cell migration in the absence or presence of hydrogel treatment at 0, 6, and 12 h. Scale bar: 100 μm. The dashed lines indicate the edges of the scratch. (**b**) Quantification of cell migration rates.

Cell migration capacity serves as a critical parameter for assessing the efficacy of hydrogels in tissue repair [43]. In this study, the cell scratch wound-healing assay was employed to evaluate the effect of hydrogels on cell migration. Representative images of cell migration were captured at 6 h and 12 h post-treatment (Figure 10a). As depicted in Figure 10b, the OPd hydrogel-treated groups maintained progressive wound closure over time without an obvious inhibitory effect on fibroblast migration. These results indicate that the OPd hydrogels do not interfere with the migratory behavior of fibroblasts and exhibit favorable biocompatibility, thus meeting the basic requirements for wound-healing materials. Taken together, the cytocompatibility and migration results demonstrate that the OPd hydrogels can support a cell-compatible environment, complementing their antibacterial, antioxidant, adhesive, and mechanical functions for potential wound dressing applications.

## 4. Conclusions

In summary, a series of multifunctional oxidized chondroitin sulfate/polyethyleneimine (OPd) hydrogels was successfully fabricated via a Schiff base crosslinking strategy. The resulting hydrogels possessed highly interconnected porous networks and high swelling capacities, while displaying typical shear-thinning behavior and good injectability. Furthermore, the materials demonstrated broad-spectrum adhesion to various abiotic materials and biological tissues, alongside favorable compressive structural stability. Biological evaluations further confirmed that the hydrogels exhibited potent antibacterial activity, notable antioxidant capacity, satisfactory hemocompatibility, and favorable cytocompatibility, effectively promoting cell proliferation and migration. Collectively, these comprehensive properties position the OPd hydrogels as promising candidates for advanced wound dressing and tissue regeneration applications.

## Figures and Tables

**Table 1:** Reagent dosages for preparing oxidized chondroitin sulfate (OCS) with different degrees of oxidation.

Sample	CS (g)	NaIO_4_ (g)	Deionized Water (mL)
OCS1	4.0	3.9	50
OCS2	4.0	4.9	50
OCS3	4.0	5.9	50

**Table 2:** The detailed compositions of OCS/PEI hydrogels.

Sample	Oxidation Degree of OCS	OCS (mg)	PEI (mg)	PBS (PH = 7.4) (mL)
OPd1	OCS1	50	100	1
OPd2	OCS2	50	100	1
OPd3	OCS3	50	100	1

## Data Availability

The original contributions presented in this study are included in the article. Further inquiries can be directed to the corresponding authors.
